# The Clinical Study of Blood-Pressure

**Published:** 1904-10

**Authors:** 


THE CLINICAL STUDY OF BLOOD-PRESSURE. By Theodore C. Janeway, M. D., Lecturer on Medical Diagnosis, University and Bellevue Hospital Medical College; Visiting Physician to the City Hospital, New York City. Seventy-five illustrations. Sold only by subscription. Octavo, cloth, $3. D. Appleton & Company, 436 Fifth Avenue, New York.

He utters a truism when the author says: "In medicine, accuracy of observation is the first step towards a correct diagnosis, without which nothing but bald empiricism is possible." A correct interpretation of the blood pressure in disease is of the highest importance to a clear understanding of a case and in prognosis; also as a valuable indication for treatment. The sphygmomanometer is an instrument of precision and can be depended upon to register the most delicate degrees of blood pressure. It is to this what the thermometer is to temperature. Dr. Janeway has made a valuable contribution to clinical study—although the multiplication of books is somewhat of a burden; a doctor can't read everything.

The work is divided into three parts: Physiological; Technical; Clinical, and under those heads the subject is treated in an exhaustive and very clean style. It is appropriately illustrated by seventy-five illustrations in the text and some in colors.

